# On the division of labor in the maintenance of intersubjectivity: insights from the study of other-initiated repair in Vietnamese

**DOI:** 10.3389/fsoc.2023.1205433

**Published:** 2024-01-08

**Authors:** Jack Sidnell, Hương Thị Thanh Vũ

**Affiliations:** ^1^Department of Anthropology, University of Toronto, Toronto, ON, Canada; ^2^Ðại Học Phương Ðông, Hà Nội, Vietnam

**Keywords:** division of labor, other-initiated repair, intersubjectivity, conversational analysis, social theories of Marx and Durkheim, Vietnamese

## Abstract

Few ideas have figured more centrally in the history of social theory than that of the division of labor. Here we ask whether conversational interaction, like other forms of social activity, exhibits a division of labor and, if so, what functions this serves and how it might be understood in relation to the theories of Marx and Durkheim. We begin by noting that, though conversational participants actively work to achieve and sustain understanding, much of the time this work is invisible and only its products are displayed in the form of sequentially fitted next turns at talk. However, in sequences of other-initiated repair, the work involved in the maintenance of intersubjectivity rises to the surface. On these occasions, we can see and thus describe what participants do to achieve and sustain what they take to be adequate understanding. In our data, which consist of video recordings of casual conversations among Vietnamese same-generation peers, participants continuously display an orientation to relations of relative seniority through the selection of terms used to accomplish interlocutor reference. This pervasive orientation is also reflected in practices of repair initiation. Specifically, seniors regularly initiate repair with so-called “open class” forms such as “huh?” and “ha?” which display a minimal grasp of the talk targeted, require little effort to produce and, at the same time, push responsibility for resolving the problem onto the trouble source speaker (i.e., the junior member of the dyad). In contrast, juniors often initiate repair of a senior participant's talk by displaying a detailed understanding of what has been said, either in the form of a repeat or a reformulation, and inviting the senior to confirm. We suggest then that this asymmetry in the distribution of initiation practices reflects a “division of intersubjective labor”. We conclude with some thoughts on the theoretical implications of our findings and relate them not only to the theories of Marx and Durkheim but also to the writings of feminist sociolinguists who sought to describe the way in which women seem to be burdened more than men with what Fishman called “interactional shitwork.”

## Introduction

Few ideas have figured more centrally in the history of social theory than that of the division of labor (for a recent overview from an anthropological perspective, see Sanchez, 2018). In his early writings, Karl Marx theorized the division of labor in relation to processes of alienation. Specifically, workers, performing specialized, repetitive tasks that figured as isolated steps in a larger productive process, were alienated not only from that which they had a hand in producing but also from the creative activity of production itself and, ultimately, their own “species being” (Marx, 1977[1844]). The later Marx (1992[1867]) emphasized a distinction between the social division of labor and the division of labor in production (Mohun, 1983). The social division of labor consists of the various ways in which labor is distributed within a society between, for instance, men and women, young and old, peasants and feudal landlords, proletariat and bourgeoisie, and so on. The division of labor in production, on the other hand, refers to the ways in which processes of production under capitalism are broken down into component operations, the prototypical example being the assembly line characteristic of factory labor. While ever more minute divisions lead to greater efficiency and increased production, for Marx and Engels this simultaneously encourages the development of social classes whose interests are fundamentally at odds. Moreover, the very conditions of labor (trade and aggregation in towns and cities under feudalism; factories and union organization in the case of industrial capitalism) lead to the development of class consciousness and, eventually, a revolutionary political movement.

In contrast to the critical perspective of Marx and Engels, Durkheim (1933[1893]) emphasized the integrative fuction of the division of labor as the primary mechanism of organic solidarity. Just as the organs of the body have specialized functions, each essential to the welfare of the whole, so too the various groupings within a modern, industrialized society make a distinct and necessary contribution to the larger collective. Buoyed by a shared set of norms, values and beliefs, the organic solidarity which emerges from the division of labor prevents the destructive forces of entropy from taking root. Despite their differences, both Marx and Durkheim believed that all societies, past and present, exhibit some kind of division of labor. In what they saw as the most primitive forms of social arrangement, this was organized along lines defined by age and sex/gender. In the 1970s, a number of Marxist and feminist anthropologists noted the apparently universal association of women with the domestic domain and with the work of social reproduction (see, *inter alia*, Ortner, 1972; Rosaldo, 1974; Godelier, 1986). They also noted an apparently near-universal denigration of this domain in relation to “public spheres that are ostensibly sites of collective dynamism” (Sanchez, 2018).

In our contribution to this special issue of *Frontiers in Sociology*, we ask whether conversational interaction exhibits, like other forms of social activity, a division of labor and, if so, according to what principles it is organized and what functions it serves.^1^ This initial statement of our aim requires some explanation and qualification. We may begin, then, by noting that intersubjectivity—shared understanding—requires effort. To put this another way, a conversation's participants actively work to achieve and sustain understanding, despite appearances that this emerges spontaneously in the turn-by-turn unfolding of talk.^2^ Much of the time, this work is invisible and only its products are displayed in the form of sequentially fitted and appropriate next turns at talk. However, in sequences of repair, and especially in sequences of other-initiated repair, the work involved in the maintenance of intersubjectivity rises to the surface. On these occasions, we can see and thus describe what participants do to achieve and sustain what they take to be adequate understanding. As such what we will describe here is not so much an “interactional” division or labor, as an intersubjective one. Our claim is that the work of maintaining mutual understanding is unevenly distributed across a conversation's participants, at least in our data.

This study responds, then, to a typically unarticulated assumption of scholarship in conversation analysis and related approaches: the idea that the work required to sustain intersubjectivity is evenly distributed among the participants, each having essentially equivalent responsibility to ensure that they are understood and that they understand others. This conceptualization fits with a pervasive egalitarian ideology that characterizes many of the settings in which talk takes place. However, there are social situations in which assumptions of egalitarianism do not hold. In our data, which consist of video recordings of casual conversations between Vietnamese same-generation peers, participants continuously display an orientation to relations of relative seniority through the selection of terms used to accomplish interlocutor reference (i.e., reference to the speaker and addressee of an utterance, see e.g., Luong, 1990; Sidnell and Shohet, 2013; Sidnell, 2019, 2022; Djenar and Sidnell, 2022). This pervasive orientation is also reflected in practices of repair initiation. Specifically, seniors regularly initiate repair with so-called “open class” forms such as “huh?” and “ha?” which display a minimal grasp of the talk targeted, require little effort to produce and, at the same time, push responsibility for resolving the problem onto the trouble source speaker, i.e., the junior member of the dyad (on trouble responsibility, see Robinson, 2006).^3^ In contrast, juniors often initiate repair of a senior participant's talk by displaying a detailed understanding of what has been said, in the form of a repeat, and inviting the senior to confirm. Not only do these practices of initiation ask little of the senior participant in terms of response and, as such, have an “assistive” feel to them, they also often mark what has been said as important, as worthy of repetition, as something that others should clearly understand and so on. We suggest then that this asymmetry in the distribution of initiation practices reflects a “division of intersubjective labor.”

## On the idea of an interactional division of labor

In her 1978 article on differences in the contributions of men and women to everyday interaction, Fishman (1978, see also 1977) concluded:

It seems that, as with work in its usual sense, there is a division of labor in conversation. The people who do the routine maintenance work, the women, are not the same people who either control or benefit from the process. Women are the “shitworkers” of routine interaction, and the “goods” being made are not only interactions, but, through them, realities.

Fishman's findings were, however, largely impressionistic and the analysis was based on an, at the time, common assumption that the functional value of a conversational “act” or “action” is the same across different sequential contexts. For instance, Fishman quantified the number of questions asked by the male and female participants in 7 h of interaction in a domestic setting. She similarly compared “minimal responses” and “statements” which “display an assumption on the part of the speaker” that they will be understood and of interest, and elicit response from their recipients. Subsequent attempts to replicate Fishman's findings failed (see McMullen et al., 1995) suggesting that, while the initial intuition of a division of interactional labor may be valid, particularly in the setting that Fishman studied, the analytic categories she employed were not sufficiently well-defined to adequately capture it.

Research on the organization of interaction done since the 1970s allows for a refinement and rethinking of Fishman's study (see, *inter alia*, Heritage, 1984; Moerman and Sacks, 1988; Sidnell, 2014). Specifically, we know that the maintenance of shared understanding or intersubjectivity requires effort. Much of the time, the work that participants do to achieve such understanding is invisible to analysts and only its products in the form of appropriately responsive next utterances are available to us. However, when they encounter troubles of understanding, conversationalists routinely employ practices of repair in their attempts to resolve them. This makes the work of maintaining intersubjectivity available for analytic inspection.

In what follows, we explore this work in a study of Vietnamese conversation. More specifically, we examine various practices of repair initiation and track their distribution across senior and junior interlocutors. This is made possible by the fact that Vietnamese conversationalists are pervasively oriented to locally relevant relations of seniority. Their *in-situ* orientation to such relations is displayed, most prominently, in the terms they use for interlocutor reference, that is, reference to speaker and addressee.

Our analysis challenges a basic assumption of work in conversation analysis—that participants in a conversation bear essentially equivalent responsibilities for the work involved in maintaining shared understanding. That assumption may be warranted in many of the settings that conversation analysts have studied—such as interaction among English speaking peers in informal conversation—but does not accurately reflect the socio-cultural realities within which Vietnamese conversation takes place. In this latter setting, relations of seniority and the different expectations in terms of interactional conduct to which they are indelibly linked, shape conversational organization in a range of significant ways.

The results of our study, and the intellectual motivation that animates it, resemble those of Ochs (1982, 1984) who, in research conducted in the early 1980s, compared what she called clarification strategies in White Middle Class American (WMC) and Samoan households. Ochs drew on work by Schegloff and other conversation analysts which seemed to show that (1) repair initiation practices exhibit a “natural ordering” based on their relative power to locate a repairable (Schegloff et al., 1977, p. 369) and (2) “speakers show a preference for using the strongest form they can in initiating repair of another's utterance” (Ochs, 1984, p. 331). Ochs found that in the Samoan context, practices of repair initiation (or what she calls “clarification”) are differentially employed depending on the relative rank of the participants:

In speaking to those of lower rank, higher ranking persons are not expected to do a great deal of perspective-taking to make sense out of their own utterances or to make sense of the utterance of a lower ranking interlocutor. Higher ranking persons, then, are not expected to clarify and simplify for lower ranking persons (…) And exactly the reverse is expected of lower ranking persons. Lower ranking persons take on more of the burden of clarifying their own utterances and the utterances of higher ranking interlocutors.

In the Samoan context, high-ranking conversationalists typically request clarification using a minimal grasp strategy (i.e., open class repair initiators) rather than an expressed guess, as the latter requires one to more obviously take alter's perspective. In what follows we will show that, in Vietnamese conversation, we find a similar pattern in which seniors tend to initiate repair with open class initiators which (1) do not require that the speaker attempt to recover what the other has said, (2) suggest that responsibility for the encountered trouble lies with the trouble source speaker (i.e., the more junior interlocutor), and (3), require little articulatory effort for their production (this itself serving as a sign of the senior participant's low level of involvement in the junior participant's talk). At the same time, we find that junior participants rarely employ such open class repair initiators. Juniors instead show a marked tendency to use a practice of repair initiation that involves repeating a more senior participant's talk with an appended question particle. Even more striking, we find that junior interlocutors engage in an apparently distinctive sequence that involves asking a senior participant a question, receiving an answer and then requesting confirmation of that answer with a repeat appended by a question particle. This practice seems to illustrate the more general tendency of juniors to carefully reconstruct and publicly check their understanding of a senior participant's talk.

## Data and methods

The data used in this study come from a larger investigation of other initiated repair and intersubjectivity in Vietnamese conversation. The corpus, collected in various coffee shops and restaurants in Hanoi in 2012, consists of approximately 35 hours of video recorded conversation among same-generation peers. For the present study we sampled five of these recordings. We summarize their basic features in Table 1. All instances of other initiated repair were collected from a portion of each recording (VNR 05 and VNR 20/21 = +/−50 min, VNR 10, VNR 12, and VNR 32 = +/−10 min). The result was a collection of 96 instances. The authors of the current report relistened to all these cases and discussed them in some detail. As we did this, we also sorted the examples into sub-collections according to the format used in the initiation of repair (see the next section for an overview). Once all the cases had been sorted, they were retranscribed and checked again, a process that resulted in additional observations.

**Table 1:** Overview of data sources and cases used in the present study.

	**Number of participants^*a*^**	**Sex and age of participants (L-R in image)**	**# of cases of repair**
VNR 05 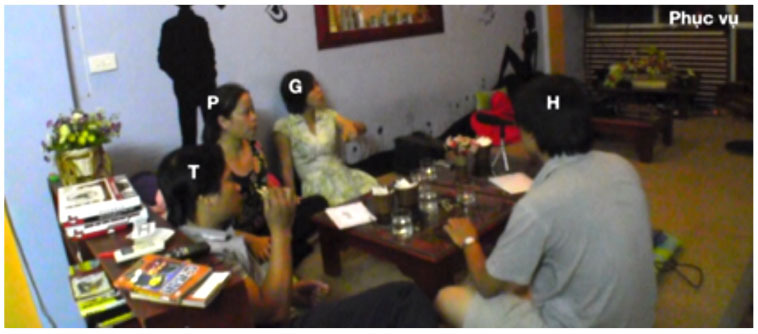	4	M33, F29, F27, M30	31
VNR 10 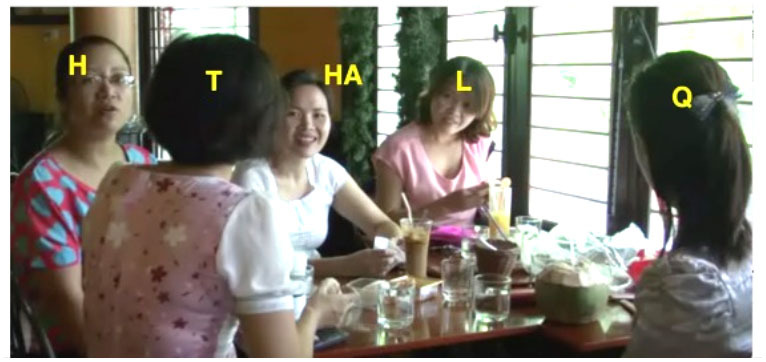	5	F34, F35, F31, F31, F34	22
VNR 12 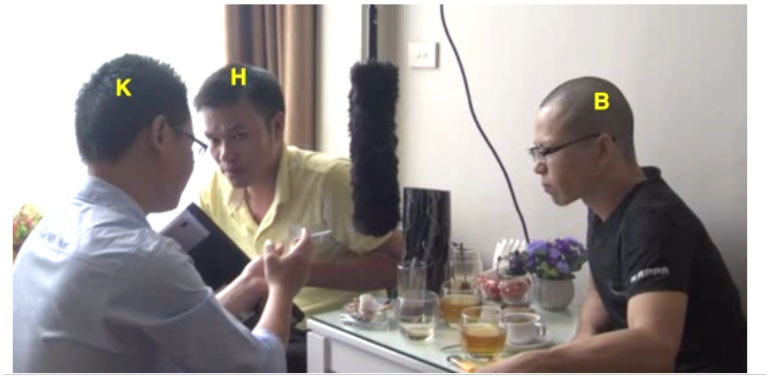	3	M25, M30, M30	22
VNR 20 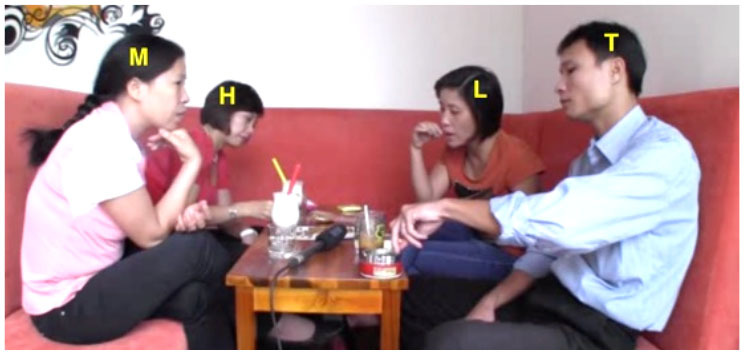	4	F45, F42, F40, M41	15
VNR 32 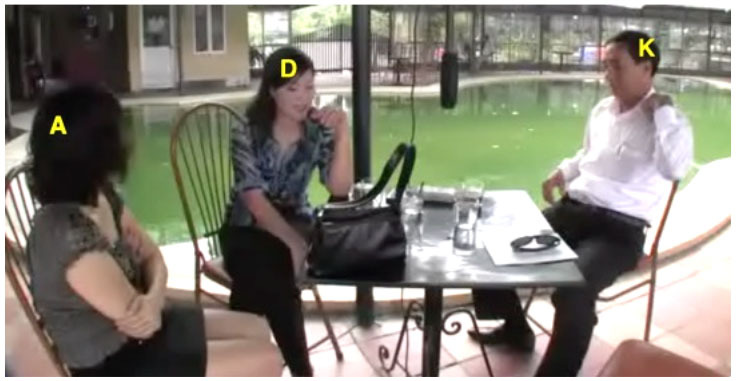	3	F47, F48, M54	6
Totals	19		96

^*a*^This does not include restaurant servers or researchers.

## Overview of repair initiation practices

Episodes of repair are composed of parts. A repair initiation marks a disjunction with the immediately preceding talk while the repair itself constitutes an attempted solution to a problem. That problem, the particular segment of talk to which the repair is addressed, is called the trouble source.^4^ Our discussion in what follows focuses on the alternative formats used in the other-initiation of repair and some of the sequential consequences that flow from the selection of one format or another. In their classic paper on the preference for self-correction, Schegloff et al. (1977, p. 367–368) distinguished five common repair initiation formats in English conversation: (1) interjections and questions words such as *huh?* and *what?*^5^; (2) question words such as *who, where, when*; (3) partial repeats of the trouble-source turn, plus a question word; (4) partial repeats of the trouble-source turn; and, (5) candidate understandings of a prior turn. In an important recent study, Dingemanse et al. (2014, p. 5) find that different languages make available “a wide but remarkably similar range of linguistic resources” for the other initiation of repair. According to these authors, alternative formats can be differentiated along a number of dimensions including the extent to which they characterize the trouble, the way they manage responsibility for the trouble, and what they imply about the relative distribution of knowledge among the co-participants.

Drawing on the distinctions introduced by Schegloff et al. (1977) as well as some terminological and analytic refinements introduced by Dingemanse et al. and others (e.g., Drew, 1997), we were able to sort the Vietnamese cases into five categories as shown in Table 2.^6^

**Table 2:** Distribution of formats used in the other-initiation of repair.

	**Open class**	**Closed class Q word**	**Repeat + Closed class Q word**	**Candidate understanding**	**Repeat (+ Q particle)**	**Total^*a*^**
N	20	4	8	25	30	87
%	23	5	9	29	34	100

^*a*^We also collected nine cases of correction which brought the total to 96 as indicated in Table 1.

Open class and repeat-formatted initiations will be examined in some detail in the discussion that follows. Here we give examples of the other formats for illustrative purposes.

In (1) the participants have been complaining about a rise in the cost of vegetables and about the dismissive attitude of those who sell them in the street markets. In line 79, Phuong, remarks that dill leaf has risen in price to 10,000 dong a bunch.^7^ Thanh, apparently unsure of what Phuong is referring to, initiates repair with *cái gì* ‘what' thereby targeting a noun phrase in Phuong's previous utterance that refers to something other than a person or a place (see footnote 5). Phuong provides a repair solution in the form of a repeat of the noun phrase (*thì là*).

(1) Closed Class Question Word (VNR05, 28:30)^8^
       79 P: *Thì là sắp lên mười nghìn rồi*.
       dill about up ten thousand already
       Dill is about ten thousand.
       (…)
       82 T: *Cái gì*.
       CL Q
       What?
       83 P: *Thì là*.
       dill
       Dill.

Below, (2) illustrates the use of a closed class question word appended to a partial repeat of prior talk. Hoàn and Ba, along with Kiên, jointly own and run a computer software and programming company. Where the extract begins, Hoàn is asking about one of several ongoing projects referring to this as, in line 07, *dự án ba* ‘project three'. Ba responds with an open class repair initiator which merely indicates a problem with the immediately preceeding turn but does not specify a particular component or aspect of this as the trouble source. Hoàn continues in line 09 apparently assuming that the problem will resolve itself as the talk progresses, but Ba initiates repair again now using a combination of repeat (*dự án* ‘project') and question word (*nào* ‘which'). Hoàn then provides a repair in line 11 saying, *Dự án đấy. Bank đấy* ‘That project. That bank'.

(2) Closed Class Question Word + Repeat (VNR12)
       07 H: *Dự án ba thế nào, triển khai đi*
       project three what about implement PRT
       What about project three, implement it.
       08B: *Ha?*
       huh
       Huh?
       09 H: *Rút ra anh em sang làm*
       withdraw EB YS cross work
       Take the project out, we will work on it.
       10 B: *Dự án nào*
       project which
       Which project?
       11 H: *Dự án đấy. Bank đấy*
       project that bank that
       That project. That bank.
       12 B: *Triển khai đi, để làm nó*
       implement PRT let's do 3
       Let's implement it, let's do it
       13 *chạy ổn định phát là ném lên ap store*
       run stable transmit throw up app store
       once it runs stably, put it up on the app store.

Finally, in (3) we see the use of a candidate understanding to initiate repair. This is taken from the same recording as example (2). Here Hoàn, Ba and Kiên are discussing how much of the company money is being spent on their various projects. The extract begins with Kiên saying that Ba has recently withdrawn 100 million dong from a company account. Ba initiates repair in line 12 and Kiên repeats in part what he has just said. Ba then responds, suggesting that Kiên has misunderstood, and that he's talking about something else, leading Hoàn to initiate repair with a form which invites Ba to confirm a proposed candidate understanding of his talk. In the first of these candidates, Hoàn proposes *Khoản đấy bỏ ra à* ‘You excluded that amount' and in the second, at line 18-19, he suggests *Nghĩa là bên kia nó nó đấu tư về à* ‘Meaning the other part is what they invested?'

(3) Candidate Understanding (VNR12, 5:40)
       11 K: *Vừ- Vừa rồi mới lấy một trăm triệu cơ mà*.
       ju- just already take one hundred million PRT PRT
       But you just got one hundred million!
       12 B: *Hmm*
       Hmm?
       13 K: *Lấy về một [trăm triệu*.
       take about one hundred million
       You took about one hundred million.
       14 B:           *[Không, không nói khoản đấy*
                 NEG, NEG say amount PRT
                 No, no, I'm not talking about that.
       15 H: *Khoản đấy bỏ ra à*
       amount there excluded PRT
       You excluded that amount?
       16 B: *Mhm, khoản kia là khoản*
       yes amount that is amount
       Yes, that's the amount
       17 *thiết bị máy móc*
       equipment machinery
       for their equipment.
       18 H: *Nghĩa dư vậy là bên kia*
       meaning like that is side there
       Meaning the other part
       19 *nó nó đấu tư về à*
       3 3 invest PRT
       is what they invested?
       20 B: *Hả? Ðâu, mình vay*
       Q NEG SF borrow
       What? No, we borrowed that!

With respect to the initiation formats illustrated by examples (1), (2), and (3), there were no clear distributional differences according to the relative seniority of the participants.^9^

## Operationalizing “seniority”

Our analysis focuses on the relation between the practices of other-initiated repair (and in particular on the use of alternative formats for initiation) and the relative seniority of the participants. Initial review of the recordings, along with native-speaker intuition, suggested that interjections (such as *huh?* and *ha?*) were used only when a senior participant initiates repair of a junior participant's talk. In addition, a slightly more sustained examination of the recordings seemed to indicate that repeats were more often used, and used in a particular way, by junior participants to initiate repair of a senior participant's talk.

In order to develop an analysis that might provide empirical grounding for these observations, we needed to operationalize a notion of “seniority.” This is an aspect of social organization toward which Vietnamese conversationalists are pervasively oriented since in almost any context a speaker must take such relations into account in designing a situationally appropriate utterance. This is seen most obviously in the terms used for interlocutor reference. As is well-established in the sociolinguistic and linguistic anthropological literature, the default means for accomplishing interlocutor reference in Vietnamese across a very wide range of contexts involves the use, not of pronouns, but rather of kin terms.^10^

Moreover, in Vietnamese there are no reciprocally used kin terms and, as such, interlocutor reference by such means results in a continuous display of relative seniority.^11^ For instance, a speaker may self-refer using a term such as *anh* ‘elder brother' or *chị* ‘elder sister' while referring to the addressee as *em* ‘younger sibling'. These relations of seniority cannot be read directly from the ages of participants for several reasons, some of which are important to the analysis of repair initiation that follows. First, if two persons are born in the same calendar year, they may consider themselves true peers and avoid the use of sibling terms that necessarily convey relative seniority. Second, in some contexts and in some social relations, relative seniority is exaggerated whereas in others it is understated. Specifically, a difference of 5 years may be treated as significant in one dyad but not in another.^12^ For these reasons, in order to operationalize seniority, we can't simply correlate some particular aspect of the speech behavior with the relative ages of the participants. Rather, we have to look at the ways in which the participants themselves orient to such relations, for instance in their practices of interlocutor reference, and use these orientations as a guide to understanding other aspects of their conduct.^13^

### Open class repair initiation

Our collection included 20 cases of open class repair initiation (see Table 3). In open class repair initiation, a speaker indicates that there is a problem with the immediately preceding turn (or TCU, see Robinson, 2014) but does not locate some particular item or aspect of it as the trouble source. Of these 20 cases, 18 involved the use of an interjection (e.g., *ha?*) while just two involved the use of a question word. In total, 13 of the 18 cases of open class repair initiation with an interjection were addressed by a senior toward a junior co-participant. In two, the relation was reversed and in three cases speaker and recipient treated one another as true peers by avoiding the use of kin terms.^14^ It is also worth noting that in two of the recordings sampled there were no instances of this repair initiation format.

**Table 3:** Distribution of two formats for open class repair initiation.

	**Senior➔Junior**	**Junior➔Senior**	**Not applicable**
Interjection	13	2	3
Q word	1	1	0
TOTAL	14	3	3

The example presented as (4) illustrates the use of an interjection to initiate repair. Here (senior, male) Thanh and (junior, female) Phuong have been talking about a time that they went together, along with Giang, to sing karaoke in Ho Chi Minh City. Thanh asks Phuong to guess how much it cost and, after some talk in which Phuong indicates that Thanh already told her how much it was, she produces the turn in line 03-04.

(4) Open Class - Interjection (VNR05, 25:07)
       03 P: *Ở đấy tám mươi nghìn*
       LOC there eight ten thousand
       It is eighty thousand
       04 *một tiếng đúng không*
       one hour correct Q
       per hour there, right?
       05 T: *Ha?*
       Huh
       Huh?
       06 P: *Tám mươi nghìn*
       eight ten thousand
       Eighty thousand
       07 *một tiếng đúng không*
       one hour correct Q
       per hour there, right?
       08 T: Ừ.
       Yes
       Yes.

Here then the senior co-participant initiates repair of the junior co-participant's talk using an interjection that does not indicate which aspect or component of the immediately preceding turn is the trouble source. In attempting to resolve the problem, the speaker of the trouble source produces a near-exact repeat of her turn, one that preserves not only the informational content of the prior talk but also its status as a polar interrogative.

The other open class repair initiation format involves the use of the question word *cái gì?*.^15^ For instance, in the following case, Hà has been telling the others about an awkward exchange she had with their superior at work. This involved inviting the superior (Hiền) to a party to celebrate Hà's daughter's acceptance to a prestigious college. This was made awkward, in the first place, by the fact that Hiền also has a daughter of the same age, whom, the co-participants surmise, had not been similarly successful with her applications. But the awkwardness was exacerbated when Hiền asked Hà whether she expected the party-goers to pay money, which is to say give a gift of cash to Hà's daughter. Hà's talk about these matters has been directed primarily to Tiến while Mai and Lệ have been occasionally talking between themselves. Here, however, Mai has, at line 06, asked Hà whether Hà told Hiền the reason for the party when she invited her.

(5) Open Class - Question word (VNR20, 23:47)
       05 M: *Lúc em mời chị Hiền*
       time YS invite ES Hien
       When you invited Hien,
       06 *em có nói lí do không*.
       YS Q say reason Q
       did you tell her the reason for the party?
       07 H: *Em không nói lí do*,
       YS NEG say reason
       I didn't tell her the reason,
       08 *nh*ư*ng ch*ắ*c chị hi*ể*u ngay*,
       but certainly ES understand immediately
       but I guess she understood right away,
       09 *chị lại bảo chứ*,
       ES PRT say PRT
       she said,
       10 *thế nào*[:: ( )
       how
       How
       11 M:       [*Ngại thế nhở*
              awkward how
              How awkward
       12 H: *Ngại thế. Sao em lại thế nhở*.
       awkward how why YS PRT like that
       So awkward! “Why did you do that?
       13 *Tiền nong như thế nào đây*.
       money insert like that here
       Just to talk about money like that!”
       14 *Có phải đóng tiền không*.
       Q must pay money Q
       “Should we pay money?”
       15 M: Ơ.
       Oh
       Oh
       16 H: *Chị hỏi em câu     đấy đấy*
       ES ask YS sentence PRT PRT
       She asked me that question.
       17 M: *Cái gì?*
       CL Q
       What?
       18 H: *Em bảo chị hỏi em*
       I say ES ask YS
       I said, “You asked me
       19 *có phải đóng tiền không*,
       Q must pay money Q
       ‘must we pay money?”'
       20 *em bảo sao dạo này*
       YS said why time this
       I said, “why are
       21 *chị kém cái độ lãng mạn đi thế*.
       ES less CL degree romantic PRT PRT
       you being so insensitive these days?”
       22 M: *Thật á*
       true PRT
       Oh really?

Three observations about this case are the following. First, although the turn in line 17 clearly initiates repair, it does this not by means of an interjection but rather with a question word, *cái gì?* ‘what?'. Second, this is produced with a marked and exaggerated prosody and in this way not only initiates repair but also conveys Mai's surprise. Third, the repair itself in line 18-19 involves not just repeating the reporting frame but also substituting direct reported speech for the indexical expression used in line 16 (*câu đấy **→**có phải đóng tiền không*).

These open class repair initiation formats are equivalent in the sense that they do not locate a particular aspect or component of the prior talk as the source of trouble (see Ochs' “minimal grasp” description). Moreover, by not attempting to fix the problem, the one initiating repair in this way seems to push the responsibility for this on to the trouble source speaker. Indeed, the default assumption appears to be that responsibility for the trouble lies with its speaker and these formats do nothing to defease an inference based on such an assumption.^16^

Beyond these basic similarities, the question-word format requires more articulatory effort than does the interjection (see Dingemanse et al., 2013; Enfield et al., 2013). The interjection consists of a single syllable and is composed of a mid, central vowel and a consonant produced with minimal obstruction of the throat and mouth. Furthermore, the interjection has no stable, context-independent semantic meaning. In comparison, the question-word format is two-syllables and is composed of two lexical segments (*cái* is a general classifer*, gì* is a question word equivalent to English “what”).

These two formats thus differ in terms of what Peirce described as the material qualities of the sign. Specifically, production of the question word requires slightly more effort and thus can potentially convey more (other-) attentiveness than the interjection. More importantly, the question word format is more amenable to modulation by intonation allowing for the display of, for instance, “surprise” and “astonishment” (see Ha and Grice, 2017). For these reasons and others, the two formats are not always interchangeable or equivalent. The distributional skewing is apparent only in the interjection-based OCRIs where we find that 13 of 15 (or 86.6%) instances are addressed by a senior toward a junior participant.^17^

We can extend our analysis and provide further evidence for it through consideration of a non-conforming case. In (6), below, the three young men have been talking amongst themselves when the (female) server sits down, off camera, at a nearby table. Kiên looks over, gazing in her direction for a few seconds before producing the talk in line 01. Taking notice of the server's t-shirt, upon which are pictured two large bird wings, Kiên asks whether, when wearing this shirt, she can fly. The question is based on a noticing of a feature of the setting which has, to this point, not been a focus of attention. Not surprisingly, then, the server, whom Kiên addresses as *em* ‘younger sibling', initiates repair. The situation in some sense demands open class repair initiation since what is at issue is the action that Kiên means to be doing, this coming out of “left-field” with little if any common ground having been already established (on this use of open class repair initiators, see Drew, 1997; Sidnell, 2010b, p. 122–124).

(6) Open Class – interjection (VNR12, 2: 15)
       01 K: *Mặc áo đấy có bay được không em?*
       wear shirt that Q fly achieve Q YS?
       Wearing that shirt you can fly?
       02 N: *Dạ?*
       Yes?
       03 K: *Mặc áo đấy có bay được không*.
       wear shirt that Q fly achieve Q
       Wearing that shirt you can fly?
       04 N: *Sắp bay được*.=
       about fly achieve
       Just about to fly.
       05 K: =*Hi-hi-he-hhhh-heh-hehe-hehe*

What we want to notice here is that the server, who is addressed as junior with *em* ‘younger sibling', initiates repair not with *ha?* as Thanh did in (4) above, or with *cái gì?* as Mai did in (5) but rather with *dạ?*. In addition to its use as a repair initiator, this form is also used to convey deference to the addressee (i.e., as a “respect particle,” see, e.g., Thompson, 1987; Shohet, 2013).^18^ Thus, we find that in one of the rare instances that a junior uses an open class format to initiate repair of a senior participant's talk, they do so by means of a particle that is understood to convey deference to the addressee.

### Initiating repair with a repeat^19^

An open class repair initiation, whether formed with a question word or an interjection, does not identify a specific aspect or component of the prior turn as the trouble source. Rather, it merely signals a problem and leaves it to the speaker of the trouble source to determine what is required for its resolution. In contrast, a repeat-formatted repair initiation identifies very precisely that part of the prior talk that is being treated as a source of trouble (see, *inter alia*, Jefferson, 1972; Hayashi et al., 2013). Moreover, when a participant initiates repair in this way they take on almost all of the work needed to achieve resolution. The speaker of the trouble source is merely asked to confirm or disconfirm.^20^ For these reasons, repair initiation in this mode can appear solicitous, even obsequious. Consider the following case (7) in which the participants, all of whom work at the same health insurance company, are talking about a time that Tiến hosted a gathering at his house which is some distance from Hanoi. Mai, the oldest person in the group, is explaining, in line 05, that she was busy that day and so couldn't come. By gazing at Tiến while she says this, Mai indicates that she is addressing him specifically with her talk. However, although Tiến does appear to produce some response (barely audible on the recording), it is Lệ who is most active in taking up Mai's talk. Thus, in overlap with the last word of Mai's turn, but at a point where it is surely projectable, Lệ repeats *Chị không sang được* ‘You [elder sister] didn't get to come' (thereby addressing Mai as *chị* ‘elder sister'). While produced with no appended particle, the repeat clearly invites confirmation from Mai by virtue of the epistemic asymmetry it indexes. Mai, who is still gazing at Tiến as she completes her turn in line 05, first acknowledges Tiến's contribution with a slight head nod (line 08) and then, shifting her gaze to Lệ, responds to the repeat repair initiation again with a brief responsive and confirming head nod (line 08-09).

(7) Repeat (VNR20, 20:32)
       03 T: *Chả muốn sửa*
       NEG want fix
       I don't want to fix it.
       04 M: *Hôm sang nhà Tiến chị bận cái gì này*,
       day come house Tiến ES busy CL Q PRT
       The day that you had people over,
       05 *nên chị không sang [được*.
       so ES NEG come get
       I was busy so didn't get to come
       06T:       [( )]
       07L:      [*Chị không sang được*,=
            ES NEG come get
            You didn't get to come
       08 M: = *Mhm* = ((M begins while gazing at T, starts to shift
       gaze toward L, while continuing to nod.
       M & L achieve momentary mutual gaze.))
       09L: =*Mm* ((L nods - composed of slight upward
       movement then down toward table,
       gaze fixes on bowl.))
       10 H: *Em mời lần nữa đi*
       YS invite time again PRT
       Invite us sisters one more time
       11 *cho các chị sang*,
       give PL ES come
       so we can visit,
       12 *khổ,        chị Dung cũng không được sang*
       unfortunately ES Dung also NEG get come
       Dung also didn't get to come.

In a case like this, there's little sense of any *actual* problem of hearing or understanding. Rather, the repair initiation seems more “assistive.” Mai is making an excuse and Lệ, by initiating repair with a confirmation requesting repeat, appears to support this effort.

Consider also the case presented as (8). Here the student research assistant who filmed the interaction (X) has been adjusting some of the equipment and, at line 57, announces that he will be sitting in the lower area of the restaurant while the video is recording, referring to himself as *anh* ‘elder brother' in doing so. After a slight pause the assistant seems ready to continue speaking but Hiền initiates repair by repeating what he has said and appending a question particle (à). The assistant confirms with Ừ, an affirmative response particle or interjection that is considered appropriate with junior interlocutors.

(8) Repeat (VNR10, 2:12)
       56 X: Rồi! mấy chị em cứ ngồi.
       there PL ES YS just sit
       Ok then! You ladies just sit here.
       57 *Anh ngồi dưới tầng một (1.0)* hh
       EB sit below floor one
       I will sit downstairs.
       58 H: *Anh ngồi dưới tầng một à*
       EB sit below floor one PRT
       You will sit downstairs eh?
       59 X: Ừ
       Yes
       Yes.

What we see in these cases then is that, coincident with a displayed orientation to asymmetrical status relations, participants in these conversations routinely use a repeat-formatted repair initiation not to deal with any obvious problem of hearing or understanding (after all they hear/understand well enough to be able to repeat the prior talk essentially verbatim) but rather to support or assist a senior interlocutor. What junior interlocutors do with these repair initiations, it seems, is to show a more senior person that they have been heard and understood. There is no sense, across the various cases collected, that the “sense” or “meaning” of the speaker's repeated words is being questioned or challenged and so on (see Robinson and Kevoe-Feldman, 2010; Sidnell, 2010b; Robinson, 2013). But neither are these repeated bits of talk being merely “registered” (see Persson, 2015).^21^

Even more remarkable are cases involving a specific sequence in which the junior participant asks a question, receives an answer and then initiates repair of the answer-turn by repeating some portion of it and appending a question particle. In doing so, the junior participant treats the senior participant's talk as something important and worthy of extra attention. Consider the following case in which Giang asks Phuong if she is planning to return to her natal village the following day. After the question is asked, there is some intervening talk between Phuong and Hung about another matter and, as such, Phuong's answer to Giang is slightly displaced (and designed in a way sensitive to that displacement). Phuong's eventual answer in line 65 affirms that she will return home tomorrow. Giang then initiates repair by repeating *mai* “tomorrow” and appending the question particle à. As the two maintain mutual gaze, Phuong confirms with a subtle head nod.

(9) Repeat (VNR05, 14:40)
       62 G: *Mai chị về quê à*=
       tomorrow ES return natal village PRT
       Going home tomorrow?
       62 H: =*Ði từ lúc bầy giờ mà lên Giảng Võ*
       go from at that time PRT up Giang Vo
       If you were coming up Giảng Võ
       63 *Làm gì mà lâu thế*.
       make Q PRT long PRT
       Why did it take so long?
       64 P: *Ði: tắc đường*.
       go traffic jam
       Traffic
       65 P: *Mai chị về*
       tomorrow ES return
       Going home tomorrow.
       66 G: *Mai à*
       tomorrow PRT
       Tomorrow?
       67 (0.6) ((P and G mutual gaze, G nods slightly
       then P gives confirmation head nod.))
       68 *Hôm nào lên. Chủ nhật hay thứ hai*
       day which up Sunday or Monday
       When are you coming back? Sunday or Monday?
       69 P: *Chủ nhật. À, chắc sáng thứ hai*
       Sunday uh probably morning Monday
       Sunday. Or probably Monday morning.

So here Giang, the junior participant, asks a question and, after it is answered, seeks confirmation of the answer with a repeat-formatted repair initiation. Formally, then, this is what has been described as a post-expansion repair sequence (see Schegloff, 2007; Sidnell, 2010a). Now we might suppose that in this case the repair sequence is prompted by the intervening talk (which displaces the response in relation to the question it answers) but many of the instances we collected cannot be explained in this way. For example, consider the following in which junior Lệ asks senior Mai what she is having to drink. After Mai answers, Lệ responds by requesting confirmation with a repair initiation that combines repetition with some lexical expansion and a question particle (that is, Mai's *thạch* “jelly” is expanded to *sữa chua thạch* “yogurt with jelly”).

(10) Repeat (VNR20, 23:02)
       84 L: *Thế cái này là cái gì chị*
       so CL PROX is CL Q ES
       What is this?
       85 M: *Thạch*.
       jelly
       Jelly.
       86 L: *Cà phê thạch à-   ah:: sữa chua thạch à*
       coffee jelly PRT- ah:: yogurt jelly PRT
       Coffee jelly eh? Uh:: yogurt with jelly eh?
       87 M: Ừ
       yes
       Yes.

In this case, the junior participant (Lệ) fills out and significantly expands the senior participant's talk.^22^ Similarly, in (11), junior participant Liễu is asking senior participant Thanh where she (along with Hiền and Quý, also present) go swimming. Liễu's first attempt to pose the question in line 35 is produced in overlap with talk by Hiền and she reasks the question in line 36 now referring to the addressee and the others as *các chị* “elder sisters.” After both Thanh and Hiền respond, Liễu requests confirmation with a repeat-formatted repair initiation in line 39. This is confirmed by Hiền in line 40 (and possibly by Thanh at the same time) and Liễu subsequently acknowledges the confirmation with *ah* in line 41.

(11) Repeat (VNR10, 5:00)
       35 H: *Nó bảo tuần sau đi bơi*
       3 say week next go swim
       He said we'll go swimming next week.
       36 L: *Các chị bơi     ở đâu*.
       PL ES swim where
       Where do you all go swimming?
       37 T: *Bơi ở Ðịnh Công*.
       swim at Ðịnh Công
       We swim at Ðịnh Công.
       38 H: *Bơi     ở định công ấy*.
       swim at Ðịnh Công PRT
       We swim at Ðịnh Công.
       39 L: *Ðịnh công á*
       Ðịnh Công PRT
       Ðịnh Công eh?
       40 H: Ừ. ((head nod))
       yes
       Yeh.
       41L: *Ah*.
       ah
       Ah.

A final case, (12), illustrates the different ways in which senior and junior participants manage these interrogative sequences. Here, junior (Hoàng) Anh interrupts senior Dung's talk to ask if she will go on a day-trip that has been planned by their employer for the following day. Orienting to Dung's status as her senior, Anh asks, *Mai chị có đi không* “Are you (=elder sister) going tomorrow?” Dung answers in the affirmative and Anh then requests confirmation with a repeat-formatted repair initiation in line 33.

(12) Repeat (VNR32, 02:46)
       28 D: *Hôm vừa rồi    làm thứ bảy*
       day recent already work day seven
       Recently I worked on a Saturday
       29 *là vì tứng là*
       because thought COMP
       because I thought that
       30 *vớt [vát được một tí*
       extra get one little
       I could make some extra money.
       31 A:      [*Mai chị có đi không*
            tomorrow ES Q go Q
            Are you going tomorrow?
       32 D: *Có*
       have
       yes
       33 A: *Mai đi à* ((A nodding))
       tomorrow go PRT ((D head nod in TRP))
       Tomorrow you're going?
       34 D: *Mai Hoàng Anh đi không*
       tomorrow Hoàng Anh go Q
       Are you going tomorrow?
       35 A: *Không. Em không đi*.
       NEG. YS NEG go
       No. I'm not going.

Notice that at line 34 Dung asks the same question of Anh that Anh asked of her—i.e., whether she is going tomorrow. After Anh answers, in line 35, Dung does not request confirmation of that answer. Rather, there is a slight lull in the talk and then Anh continues by explaining that she has other plans for the day.

In these sequences of talk then, by using a repeat formatted repair initiation to request confirmation of a just given answer, the junior participant treats the senior participant's talk as something of particular importance, something that the junior participant is concerned to get “right.” At the same time, all the repeat-formatted repair initiations involve the participant initiating repair taking on more of the work than the participant who produced the trouble source. The senior participant, the trouble source speaker, is required only to confirm, typically with a minimal interjection or in many cases just a slight head nod, that which the junior participant formulates. The relative effort involved here then diagrams their different entitlements and responsibilities—A junior participant is expected to make efforts to support, to anticipate and to do their best to figure out what a senior participant means to say. A senior participant is required only to produce the most minimal kinds of confirming responses (see Wu, 2008 for a partially parallel analysis of Mandarin).^23^

We have observed, in the cases shown above, that the repair initiation seems intended to assist or support the speaker whose talk is being repeated. This appears to be a quite general and pervasive feature of the examples we collected and fits with the broader distributional pattern. In 21 of the 30 cases we collected (or 70%), it was the junior rather than the senior participant who used the repeat-formatted initiation. That said, the distributional pattern for the [repeat] + particle format is significantly more complex than for the open class interjection format we have considered above. This greater complexity is the result of two factors. First, use of an open class repair initiator is significantly constrained by a proscriptive norm which does not apply in the case of repeat-formatted initiations. Specifically, open class repair initiators are considered to be “rude” or “abrupt” and so not appropriately addressed to a recipient who is the speaker's senior. Second, in the case of repeat-formatted initiations, there are several distinct contextual configurations that provide for an appropriate occasion of use or, put another way, there are several distinct uses to which this format can be put. The numerical distribution is skewed (toward a use by juniors toward seniors) because of inferences that *may* rather than *must* accompany its use and because of the kind of interactional work it *can* but *need not* do.

Notice then that in the cases we have so far considered the use of a repeat-formatted initiation implicitly positions alter as an epistemic authority, i.e., as in a position to confirm or disconfirm that which is targeted for repair. In the cases we have examined, this epistemic authority flows, at least in part, from the fact that alter is the author of the talk upon which repair is initiated. But other epistemic considerations can easily override the importance of seniority, resulting in uses of this format by seniors to initiate repair of a junior's talk. For instance, in one case, a senior accountant uses this format to initiate repair of talk by a junior nurse about which exams are required in order to complete a university medical degree. In another case, a senior initiates repair on a junior's talk about the place that the junior's wife is currently working.

More striking are cases in which the format of [repeat] + particle serves quite different interactional ends. Whereas this format often, indeed typically, has an assistive or supportive character to it, in a small number of cases it serves agonistic ends. The fragment shown as (13) below provides an example of this somewhat unusual pattern in which a junior interlocutor is interrogated by a senior one. Here the senior interlocutor (H) is questioning the junior (L) about something construable as behavior expected of a good or pious Vietnamese woman—prayer—and uses the repeat-formatted repair initiation to do this, and specifically the Q-A-RI-C sequence we have described above.

(13) VNR_10_NTT_08_31_12_01A
       27 H: *Nhà Liễu có cúng rằm không*
       house Liễu Q pray mid-month Q
       In your house, do you pray on the full moon?
       28 L: *Không*.
       NEG
       No
       29 H: *Không cúng rằm à*
       NEG pray mid-month PRT
       Don't pray on the full moon eh?
       30 (0.2)
       31 L: *Không cúng*.
       NEG pray
       Don't pray.
       32 H: *Có cúng không*
       Q pray NEG
       Do you pray at all?
       33 L: ((shakes head, but does not look at H))
       34 H: *Không à*
       NEG PRT
       No, eh?
       35 *Thế có ăn không, sinh nhật không*
       then Q eat Q, birthday Q
       Do you eat? Birthdays?
       36 (.) *Rằm không*
       full moon Q
       Full moon?
       36L: Hi (0.2) *Sinh nhật á. Sinh nhật ai*.
       birthday PRT birthday who
       Birthday? Whose birthday?

Here then senior H uses this practice to insist upon greater explicitness by L and to treat L's answers as insufficient. Notice that insufficiency of response is conveyed in the repair initiator at line 29 by expanding the answer given, reworking this as a repeat-confirmation rather than an interjection (on the various implications attending these alternate confirmation formats, apparently cross-linguistically, see, inter alia, Heritage and Raymond, 2012; Enfield and Sidnell, 2015). The same can be said of the repair initiation at line 34 which marks the immediately preceding non-verbal response (a lateral head shake, while looking down toward the table rather than at H) as insufficient by “repeating” its propositional meaning as *không* “no” and appending a question particle (and thereby requesting confirmation).

### Two illustrative dyads

**Figure 1:**
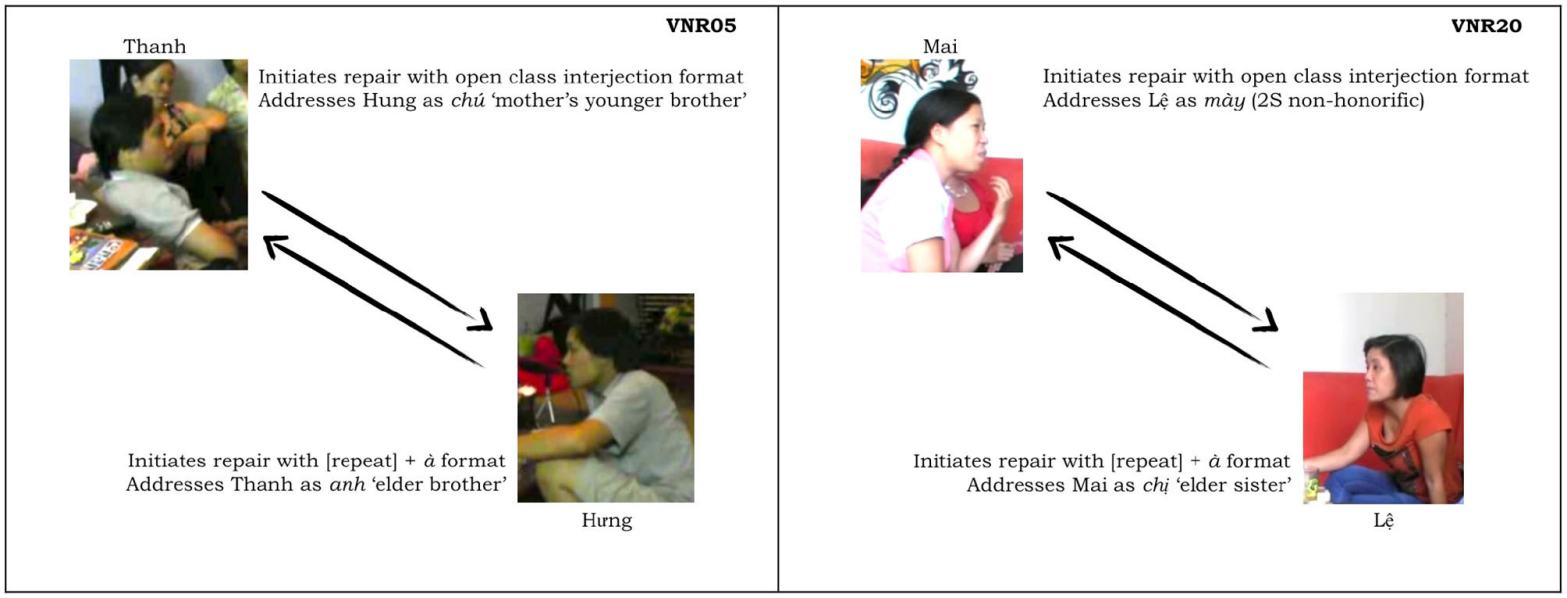
Two illustrative dyads.

Our argument about the division of labor in this domain and specifically the expectation that junior participants shoulder more responsibility for the maintenance of intersubjectivity than their senior interlocutors can be further illustrated by a consideration of some exemplary dyads (see Figure 1). For instance, in VNR 05, senior Thanh twice initiates repair of junior Hung's talk using an interjection, whereas Hung never initiates repair of Thanh's talk in this way. At the same time, Hung does initiate repair of Thanh's prior turn with a repeat-formatted initiation, while Thanh does not employ this format with Hung. This asymmetry correlates with a particular pattern of interlocutor reference in which Hung addresses Thanh as *anh* ‘elder brother' and self-refers with *em* ‘younger sibling' while Thanh addresses Hung as *chú* ‘mother's younger brother' and self-refers as *anh*. This use of *chú* involves a shift of the referential *origo* to Thanh's non-existent child and in this way highlights his own seniority vis-à-vis Hung (see Luong, 1984, 1990; Luong and Sidnell, 2020 for further discussion).

In VNR 20 a similar kind of pattern can be observed in the conduct of senior Mai and junior Lệ. Whereas Lệ several times initiates repair of Mai's talk using the repeat-formatted repair initiation in ways that, as noted, seem other-attentive if not slightly obsequious (see examples 6 and 9 and discussion thereof), Mai initiates repair of Lệ's talk with an open class interjection format. This is shown in (14) below:

(14) Open Class—Interjection (VNR20, 28:04)
       729 L: *Ơchị Dung hôm nay được làm muộn một tí à.*
       ES Dung today get do late one bit PRT
       Dung is allowed to come back a bit later, isn't she?
       730 M: *Há* ((Mutual gaze M and L))
       Huh?
       731 L: °*một giờ hơn rồi*.°
       one hour more PRT
       After one o'clock

Here then Lệ remarks, somewhat out of the blue, that a co-worker named Dung has been given permission to return late from lunch. Mai initiates repair with an interjection, and Lệ repairs the problem by specifying how much extra time Dung has been given.

What is particularly remarkable about this dyad is that while Lệ addresses Mai as *chị* ‘elder sister', Mai addresses Lệ not with *em* ‘younger sibling' but with the non-honorific second person singular pronoun, *mày*. While Mai is the oldest of the four co-participants, Lệ is the only one that she addresses in this way.

These two dyads illustrate, at the interactional level, the more general pattern visible in the aggregate. Looking at these particular cases it is possible to see the way that these practices of repair initiation (and repair generally) constitute one part of a larger set of norms that shape interaction according to the relative seniority of the participants.

## Conclusion

In a *tour de force* exploration of discursive practice and linguistic meaning in Vietnamese, Hy van Luong suggested that the pragmatic significance of person referring expressions (including kin terms, titles, names and pronouns) can only be understood in relation to competing models of and for reality (Luong, 1984, 1990, the notion of a model “of and for reality” is adapted from Geertz, 1973: 93). The pragmatic significance of kin-terms, in particular, is construable in relation to either of two contradictory models. Luong (1990, p. 50) explains:

Of the two structurally opposed models in Vietnamese kinship, one is male-oriented, and the other, non-male-oriented. One is based on the rigid separation of the sexes, and the other, on the unity of opposite-sex individuals. One has as its key unit a spatially bound but temporally unbound entity, and the other, a spatially unbound but temporally bound one. One is constructed in terms of the linear conception of time, and the other, a cyclical conception.

Construed in terms of the male-oriented model, *họ* ‘last name, family name, family' refers to a “locally based patrilineage.” Construed in terms of the non-male-oriented model this same term refers to a “bilateral kindred.” Luong further suggests that these alternative kinship-relational models “conjoin at one level and contradict each other at another.” As he writes:

… these models conjoin in that they are constructed out of the same elements (genealogy and behavioral patterns). Second, both are encompassed within an overarching organic unity framework that emphasizes, in the native metapragmatic awareness, solidarity and hierarchy among the members of the same sociocultural unit.

At this level, then, the “organic unity framework” contrasts with another possibility, which Luong refers to, drawing on the work of Turner (1974), as a *communitas* alternative. Thus, construed in relation to the organic unity framework, in either its male-oriented or non-male oriented guises, the use of pronouns *tao* and *mày*, for instance, suggests the absence (or suspension) of a relation based on kinship or any other positively valenced social relation and thus, by implication, contempt or denigration. Construed in relation to the *communitas* alternative, however, these same forms convey solidarity, extreme familiarity and even intimacy (see Zuckerman, 2023 for a similar case from Laos).

In this way, Luong recasts Durkheim's notion of organic solidarity (based on notions of differentiation and specialization within a larger whole, here a family) as a semiotically mediating ideological orientation rather that as the inevitable product of the division of labor characteristic of a particular social formation. We propose that the materials considered above fit well with this conceptualization. Specifically, in the patterns of other initiated repair here documented, we see a pervasive concern among conversationalists with relations of relative seniority and with the duties and entitlements that normatively attach to positions within an asymmetrically organized social arrangement.

Does this suggest a system of exploitation similar to that which Marx found in the division of labor associated with capitalism and which Fishman proposed could also be identified in cross-sex interactions among white middle-class Americans in the 1970s? Two features of the present case speak against this. First, relations of seniority lack the stability of class or gender relations *within a particular encounter*. For instance, a participant may be positioned as junior relative to one co-participant and as senior relative to another. If the analysis proposed here is correct, such a participant will be obligated to support the maintenance of intersubjectivity at one moment and entitled to expect such support from another at the next. Second, the relations of seniority which organize the intersubjective division of labor lack the stability of class or gender relations *across the life course*. Any given individual will find themselves gradually occupying the senior role across more and more interactional encounters as they age. For these reasons, the division of labor we have identified here seems not to be a system of exploitation *per se*, but rather an asymmetrical organization of duties and entitlements.

To conclude, our study suggests that, in Vietnamese conversation, participants are oriented to a normative division of labor which demands junior interlocutors expend more effort than senior ones in the maintenance of intersubjectivity. Specifically, whereas senior interlocutors regularly initiate repair with a form that pushes responsibility for the problem onto another participant, junior interlocutors more often initiate repair in ways that display close attention to, and detailed understanding of, a senior interlocutor's talk. In terms of larger theoretical questions, our study points to some of the complex ways in which the “social” bears on the “interactional.” We note that much research in CA that attempts to address the question of when and how perduring social facts bear on the organization of interaction focuses on participants' invocation of these facts (whether explicitly or implicitly). This approach appears to assume that the social order is brought to bear on interaction when the vernacular categories of everyday or institutional life (such as, e.g., “men” and “women,” “queer” and “straight,” “old” and “young” etc. for English) are imported into it. We have come at the problem from a different direction, and this has revealed a quite different way in which the social bears on the interactional. Specifically, beginning with participants' displayed orientations to seniority (displayed, that is, in their selection of terms for interlocutor reference), we discovered a robust correlation with the practices involved in the other-initiation of repair. We have proposed that this reflects an unequal distribution of the work involved in the maintenance of intersubjectivity. Notice then that the perduring facts of age are integrated, lockstep, with the organization of interaction. Age is not being “invoked” by the participants as relevant to the organization of interaction. Rather, the practices of interaction are, in part, organized by reference to it. But note also that the social facts (of age) which are built into these sequences are not entirely isomorphic with the vernacular categories of explicit reflection and ratiocination. Rather, “age” is integrated as a wholly indexical variable (“indexical” in the sense of Garfinkel, Sacks, and Schegloff), always calculated in relation to the age of those others with whom a given participant finds themselves interacting. The social is not, as it were, plucked from the sky and made to serve interactional ends. Rather, the social is woven into the warp and weft of interaction as it unfolds moment-by-moment, turn-by-turn.

## Data availability statement

The datasets presented in this article are not readily available because of restricted access. Requests to access the datasets should be directed to jack.sidnell@utoronto.ca.

## Ethics statement

The studies involving humans were approved by Research Ethics Review Board, University of Toronto. The studies were conducted in accordance with the local legislation and institutional requirements. The participants provided their written informed consent to participate in this study. Written informed consent was obtained from the individual(s) for the publication of any potentially identifiable images or data included in this article.

## Author contributions

All authors listed have made a substantial, direct, and intellectual contribution to the work and approved it for publication.

## Footnotes

^1^A reviewer of the paper suggests that “in the domain of repair” there have been “clear (if tacit) references to the division of labor” in such notions as “self” and “other” initiation of repair. We would suggest that references to “self” and “other” here and elsewhere in the literature point to an *interactionally* relevant division of responsibilities rooted, primarily though not exclusively, in the organization of turn-taking. This is not a “social” division of labor if “social” is taken in its usual sense, i.e., the meaning it has in the collocation “social theory”. When we say “social” division of labor, then, we mean a distribution based on organizational principles that transcend a given occasion, e.g., gender, class, race, ethnicity, age and so on. Of course, we recognize that the founders of conversation analysis (Harvey Sacks, Emanuel Schegloff, and Gail Jefferson) challenged this dichotomization of the social and the interactional by arguing that the interactional is always social (and perhaps also that the social is always interactional). While we accept and agree with these arguments, in this paper, in the interests of contributing to the theme of the special issue, we take “social” in “social theory” as it was presumably intended by the guest editors to refer to aspects of the organization of social life that are perduring and whose relevance transcends any particular occasion. This seems to be the only way it makes sense to talk about CA and “social theory” since if interaction is already social, CA is already social theory.

^2^This sense of the effortlessness of conversational intersubjectivity is presumably a result of the fact that understanding is generated, largely, *en passant* as Heritage (1984) put it. But, while understanding may emerge in the course of accomplishing the official business towards which participants are more explicitly oriented, it is nevertheless the product of a kind of labor or work as we aim to show.

^3^We recognize that responsibility for trouble is structurally tilted towards the speaker of the trouble source by virtue of the organization of turn-taking (see Schegloff et al., 1977; Robinson, 2006). The claim embodied in in our use of “push” here and elsewhere is only that OCRIs like English “what?” and “huh?” (along with their Vietnamese equivalents) do nothing to defease “structural tilt” and indeed make it more explicit. This is, however, largely irrelevant for our argument which concerns a social division of labor (“social” in the usual sense, i.e., pertaining to forms of organization that perdure beyond a given occasion of interaction, for instance groupings of “race”, “class”, “gender”, “age”) not one tied to interactionally transitory roles. Notice also that repair and understanding are hardly unique in this respect. Completing the turn-at-talk is the default responsibility of the current speaker, responding to a question is the default responsibility of the primary recipient of that question. What we are documenting thus runs orthogonal to any such a distribution of responsibilities associated with turn-taking. We thank a reviewer for encouraging us to clarify these aspects of our argument.

^4^The trouble source is to be distinguished from the source or basis of trouble, which can be anything from ambient noise, age-related hearing loss, or an esoteric word choice.

^5^In English, intonation distinguishes different types of repair initiation with a question word. Thus, *what?* produced with rising intonation typically serves as an open class repair initiation and treats an entire turn (or turn constructional unit) as the trouble source. In contrast *what*. produced with downward, final intonation serves as a closed class repair initiation by targeting some particular component noun phrase within the previous turn as the trouble source. We found a similar contrast in Vietnamese *cái gì* when used as a repair initiator (see also Ha and Grice, 2017).

^6^A reviewer asks how the “type of trouble” is related to the uneven distribution of repair initiation formats in Vietnamese and, more specifically, whether “the type of trouble (partly) explains why some of the repair initiations are unevenly distributed while others are not?” This is an intriguing possibility. A problem, though, lies in the fact that the relation between repair initiation format and trouble type is highly contingent in Vietnamese (as in all languages) and also always a matter of construal (i.e., in producing a repair initiation formatted in a certain way, the initiator thereby proposes a construal of the trouble type at issue). In an analysis of OIR in person reference, Sidnell (2007) suggests that there is a close to one-to-one mapping between a specific initiation format [“Who (is) name(d) so”] and a particular kind of trouble (the initiator believes they know the person being talked about but not by the name which has been used to refer to them). This, however, appears to be the exception rather than the rule and the case discussed involves some rather specific demographic and onomastic conditions which make certain kinds of trouble particularly common.

^7^*Ðõong* is the national currency of Vietnam. At the time of recording 10,000 dong was the equivalent of about 0.50 USD (50 cents).

^8^Transcripts are presented using a version of Jeffersonian conventions modified according to the requirements of comparative linguistic analysis (see Sidnell, 2009). Punctuation in the original Vietnamese language line represents aspects of speech prosody (e.g., a question mark indicates rising intonation, not necessarily that the utterance is a question). Punctuation in the English gloss represents an analysis of utterance meaning (e.g., a question mark indicates that the utterance is interrogative, not necessarily that it is produced with rising intonation).

^9^One reviewer suggests that the use of a candidate understanding - the most specific repair initiation type - would seem to provide a participant with an ideal means for taking up the responsibility of maintaining intersubjectivity. Why, then, the reviewer asks, are “seniors using candidate understandings as much as the juniors”? Our aim in this paper is to show that the work involved in the maintenance of understanding in interaction is socially distributed, with juniors shouldering more responsibility for this than seniors. To that end, we examine those practices which provide the clearest evidence for our claims. In order to make our case, we do not need to examine each and every practice of repair and because we did not find a robust distributional skewing in candidate understandings, we do not attempt to analyze these cases in detail. Notice that whether a given practice is caught up in the social distribution of intersubjective labor cannot be solely an effect of its function (which as the reviewer notes in the case of candidate understandings is to serve as “the most specific type of repair initiation”). For instance, it's possible that candidate understanding repair initiations are not organized by the division of labor that we show skews the distribution of some other formats because participants do not treat them as assistive to the trouble source speaker. Alternatively, or in addition, the production of a candidate understanding - which necessarily involves an at least partial reformulation of the trouble source speaker's talk- is vulnerable to being heard as an assertion of agency (essentially a claim to independent authorship, something discussed in much work on alternate responses to polar questions, see, for instance, Enfield and Sidnell, 2015, 2017). This again may affect the distribution of such practices between seniors and juniors. There is also the issue of “multifunctionality” which we discuss with respect to the [repeat] + question particle format. A given practice of speaking can, of course, be used in more than one way, and this has implications for its distribution. The [repeat] + question particle format, for instance, is routinely used by a junior interlocutor in way which appears assistive, supportive, attentive. However, as we show below, it can *also* be used in a very different way as a practice for something like interrogation, and this has implications for its distribution. Finally, there is the ever-present issue of “collateral effects” which, again, can play a role in shaping distributional patterns (Sidnell and Enfield, 2012; Enfield and Sidnell, 2017). The point, then, is that an analysis of candidate understandings would require a case-by-case consideration. This falls beyond the scope of the present report.

^10^To clarify, in Vietnamese and several other languages of the region, kin terms and other noun phrases are used to refer to speaker and addressee and as direct arguments of the verb. They are thus used in syntactic positions otherwise occupied by pronouns in many languages especially those belonging to the Indo-European family. For this reason, some linguists suggest that kin terms are themselves pronouns (see Pham, 2011 for discussion). An alternative approach describes the behavior of kin terms in these languages under the heading of “imposters” (see especially Kaufman, 2014).

^11^Although the semantics of Vietnamese kinship terms suggest that they are never reciprocally usable (e.g., the two members of a dyad cannot both be “elder brother” to the other), Sidnell and Shohet (2013) discuss one exception to the rule.

^12^A third way in which relations of seniority do not map directly from relative age is not at issue in our study but should nevertheless be mentioned. In relations between family members, seniority in the ascending (or second ascending) generation is prioritized over seniority in ego's generation such that, for example, the 13 year old son of a younger brother addresses his 11 year old cousin as *anh* “elder brother/cousin.”

^13^In Vietnamese, at least in the kinds of interactions we are considering here, speakers make relations of relative seniority explicit every time they refer to themselves or to their addressee(s). While there are conditions under which the terms can change over time (e.g., if a person becomes a parent terms of reference may change, if two persons become more intimate, they may change), in all the interactions we consider, they do not do so. This is not, then, a matter of “taking a stance”. We use the term “seniority” in its standard sense to refer to positions in an age-based and, in this case, fully-ratified system of social stratification. In sum, seniority is not something which is interactionally negotiated—it is largely a function of age, with some minor qualifications (see footnote 12).

^14^Ba and Hoàn in VNR 12 are same age peers and do not use sibling or other kin terms to address one another or to self-refer.

^15^Such uses of *cái gì* are distinguishable from closed class uses (illustrated by 1 above) by intonation, by response and, in many cases at least, by sequential position.

^16^Compare here apology-based formats for open-class repair initiation, discussed by Robinson 2006. See also footnote 2 above.

^17^We thank a reviewer for insisting on the importance of this distinction between the two formats.

^18^Thompson (1987, p. 260) describes this form as a “polite responsive particle, signalling a courteous reaction to the speech of another speaker.” Our analysis is that the speaker attempted to ward off unwanted inferences associated with OCRI by using a word that is prototypically associated with the expression of deference to alter (a good deal of effort is invested in socializing children to its use in speaking to members of ascending generations, see Shohet, 2013).

^19^Most of the cases we discuss involve the use of a repeat with an appended question particle. There is one case, 7, in which, however, there is no appended particle. This points to the fact that, under certain conditions, the interrogative character of the repair initiating turn is conveyed by means other than a particle though, in our collection, not by intonation. In 7, the repeat is marked as a confirmation requesting question by a distinctive facial expression and also by momentarily held mutual gaze (in addition to the epistemic asymmetry it presupposes). Reviewers wondered whether the repeat-formatted turns we discuss here are truly initiating repair. We maintain that whether these are characterized as repair initiators or something else makes no difference to our larger argument and indeed amounts to a binning exercise in any case (on binning, see Enfield and Sidnell, 2017). That said, there are good reasons for describing these as repair initiators if we take it that repair, by definition, involves [1] a break in “progressivity” and so a digression away from the main line of action and toward the task of fixing a problem with something already said, [2] an attempt to resolve what is treated as a problem or potential problem of speaking, hearing or understanding (as evidenced by the fact the turn in question elicits a confirming response from the trouble source speaker). Every one of our cases satisfies both these conditions. Indeed, we held the production of a confirmation (whether realized as a vocalization, a head nod or in some other way) as a strict criterion for inclusion in the collection, i.e., we only included cases in which, however obvious the meaning and significance of the trouble source utterance appeared, to us, to be, the participants treated it as, at least momentarily, in question. Finally, we want to register the fact that categorizing a given utterance as the initiation of repair does not preclude the possibility that it might ALSO and SIMULTANEOUSLY be accomplishing a wide range of other conversational “actions” such as marking the previous talk as noteworthy, important, a possible basis for further talk, and so on. Indeed, repair initiation always seems to implicate other actions or functions. Schegloff et al. (1977) and Jefferson (1987 and elsewhere) made this point in their pioneering work on the topic. One reviewer also asks whether these turns might not be analyzed as “newsmarks”. These turns do not serve to open up topics for further elaboration as newsmarks typically do. Rather, they mark what is said as potentially important by requesting that the original speaker confirm another participant's “hearing” (and by extension understanding), i.e., the part of the talk that is repeated. Notice that in all our examples, the third position confirmation closes the sequence and the talk either returns to what was being done before repair was initiated or develops in another direction (with 12 as a possible exception, but of a rather special sort). All in all, these are near-standard cases of other-initiated repair. Another reviewer suggests along similar lines that these turns might be analyzed as “acknowledging new information”, noting that they often occur in third position after a question-answer sequence and suggesting that this is a “typical location for acknowledging new information, whereas repair initiations are not tied to any sequential position.” It is true that the repeat-formatted turn does tend to occur in third position (relative to a preceding question and response/answer). However, there is a crucial difference in the cases we consider which is seen in the fact that they elicit confirmation. Note that “acknowledging new information” is not, in and of itself, interrogative i.e., it does not request confirmation that the information acknowledged was properly understood, heard etc.. The cases we consider do involve something like the “acknowledgement of new information” (we prefer to say that they mark what was said as important, noteworthy etc. since it may not always be “news”) but they do this by means of a request for confirmation that this “information” was properly understood etc.. In this they are repair initiations that make a confirming response conditionally relevant (and are unlike other third turn “acknowledgement” responses such as English “oh” which do not, or need not). In sum, “acknowledgement” does not capture the “illocutionary force” of the turns in question which is fundamentally interrogative (as are all other-initiations of repair).

^20^Indeed, confirmation is commonly given using an interjection which, like that used to initiate repair, iconically represents the limited effort that went into its production. Taking this observation further, we note that confirmation is often so minimal as to be nearly inaudible and invisible, e.g., just a slight redirection of gaze in case (6) along with an extremely subtle head nod.

^21^We did collect several cases in which repeats are used to register and to acknowledge some prior talk. Often this is accompanied by a shift in gaze away from the speaker of the repeated talk (suggesting that no response is expected). Question particles are not appended to such registering repeats.

^22^Although, it should be noted, the talk here does not distinguish this from some other menu item as “jelly” is always served with yoghurt.

^23^A reviewer asks us to clarify what we mean by “interactional effort” and specifically to say what range of phenomena we mean to include under this heading (e.g., “phonetic production”, “sequential projection”). We do not know in advance of an empirical investigation what might be included here but it seems obvious that in some basic sense producing two syllables involves more effort than producing one, and producing a repeat-formatted interrogative involves more effort than producing an injection such as *huh*? (and not just in terms of production). For these reasons, we believe that these are two aspects of the same phenomenon—one in which more senior participants are expected to expend less energy than junior participants. This resonates with widely reported cultural patterns across Southeast Asian speech communities in which relative seniority and in some cases power is associated with silence and immobility. For two classic studies that make this point in very different contexts and in different ways, see Anderson (1991) on Java and Rosaldo (1980) on the Ilongot.
